# Methotrexate area under the curve is an important outcome predictor in patients with primary CNS lymphoma: A pharmacokinetic–pharmacodynamic analysis from the IELSG no. 20 trial

**DOI:** 10.1038/sj.bjc.6605559

**Published:** 2010-02-02

**Authors:** M Joerger, A D R Huitema, S Krähenbühl, J H M Schellens, T Cerny, M Reni, E Zucca, F Cavalli, A J M Ferreri

**Affiliations:** 1 Department of Oncology and Hematology, Cantonal Hospital, Rorschacherstrasse 95, St Gallen 9007, Switzerland; 2 Department of Pharmacy and Pharmacology, Slotervaart Hospital/The Netherlands Cancer Institute, Plesmanlaan 121, Amsterdam 1066 CX, The Netherlands; 3 Division of Clinical Pharmacology and Toxicology, University Hospital, Hebelstrasse 2, Basel 4056, Switzerland; 4 Department of Medical Oncology, Antoni van Leeuwenhoek Hospital/The Netherlands Cancer Institute, Plesmanlaan 121, Amsterdam 1066 CX, The Netherlands; 5 Division of Drug Toxicology, Department of Biomedical Analysis, Faculty of Pharmaceutical Sciences, Utrecht University, F.A.F.C. Wentgebouw, Sorbonnelaan 16, Utrecht 3584 CA, The Netherlands; 6 Medical Oncology Unit, Department of Oncology, San Raffaele H Scientific Institute, via Olgettina 60, Milan 20132, Italy; 7 Oncology Institute of Southern Switzerland, Via Vela 6, Bellinzona 6500, Switzerland; 8 Unit of Lymphoid Malignancies, Department of Oncology, San Raffaele H Scientific Institute, via Olgettina 60, Milan 20132, Italy

**Keywords:** methotrexate, high-dose chemotherapy, CNS lymphoma, cytarabine, pharmacokinetics

## Abstract

**Background:**

This analysis was initiated to define the predictive value of the area under the curve of high-dose methotrexate (AUC_HD-MTX_) in patients with primary central nervous system lymphoma (PCNSL).

**Patients and methods:**

We included 55 patients with PCNSL and available pharmacokinetic (PK) data from the International Extranodal Lymphoma Study Group (IELSG) no. 20 trial, randomised to HD-MTX (*n*=30) or HD-MTX and high-dose cytarabine (HD-AraC) (*n*=25). Individual AUC_HD-MTX_ from population PK analysis was tested on drug toxicity and clinical outcome using multivariate logistic regression analysis and Cox hazards modelling.

**Results:**

AUC_HD-MTX_, the IELSG score and treatment group were significant predictors for treatment response (complete or partial) in the adjusted model. The AUC_HD-MTX_ did not predict toxicity, with the exception of liver toxicity and neutropaenia. A high AUC_HD-MTX_ was associated with better event-free survival (EFS) (*P*=0.01) and overall survival (OAS) (*P*=0.02). Both the AUC_HD-MTX_ and the IELSG score were significant predictors of EFS and OAS in the adjusted model, with a hazard ratio of 0.82 and 0.73, respectively, per 100 *μ*mol l^−1^ h^−1^ increase in AUC_HD-MTX_.

**Conclusions:**

Individualised dosing of HD-MTX might have the potential to improve clinical outcome in patients with PCNSL, even when administered concurrently with HD-AraC. In the future, this could be carried out by using first-cycle PK modelling with determination of potential dose adaptations for later cycles using Bayesian analysis.

Primary central nervous system lymphomas (PCNSLs) represent 4–6% of extranodal non-Hodgkin's lymphomas, but their incidence in the general population is increasing (Ferreri *et al*, 2003a). High-dose methotrexate (HD-MTX) is a cornerstone of PCNSL treatment (Reni *et al*, 1997; Ferreri *et al*, 2002). Only recently, the International Extranodal Lymphoma Study Group (IELSG) concluded the first randomised study in immunocompetent patients with PCNSL (IELSG no. 20). A significant increase in complete remission rate and event-free survival (EFS) was found when adding high-dose cytarabine (HD-AraC) to HD-MTX (Ferreri *et al*, 2009). Accordingly, combined HD-MTX/HD-AraC should be seen as the new standard upfront treatment in PCNSL, as it is supported by the best level of evidence available in this disease (Ferreri *et al*, 2009). Treatment with HD-MTX is hampered by a highly variable pharmacokinetic (PK) behaviour, in part related to renal elimination and the potential for drug interactions (Evans and Christensen, 1985; Thyss *et al*, 1986; Ferrazzini *et al*, 1990; Reid *et al*, 1993; Ronchera *et al*, 1993; Takeda *et al*, 2002; Joerger *et al*, 2006). However, achieving a minimum area under the curve (AUC) of HD-MTX (AUC_HD-MTX_) might be important for clinical outcome in patients with PCNSL (Ferreri *et al*, 2004). In this study, we report a PK–pharmacodynamic (PKPD) analysis of HD-MTX in patients enrolled into the IELSG no. 20 trial, to define the predictive value of AUC_HD-MTX_ and to identify clinical and therapeutic variables that could be manipulated to improve MTX efficacy in patients with PCNSL.

## Materials and methods

### Patient population and treatment

We included 79 immunocompetent patients with PCNSL, randomised to receive either HD-MTX alone (*n*=40) or HD-MTX with sequential HD-AraC (*n*=39) from the IELSG no. 20 trial (Ferreri *et al*, 2009). High-dose MTX was administered at 3.5 g m^−2^ (0.5 g m^−2^ in 15 min, followed by 3 g m^−2^ as a 3 h infusion) on day 1 in both arms, and HD-AraC was administered at 2 g m^−2^ as a 1 h infusion every 12 h on days 2 and 3 in the combined treatment arm. Radiotherapy was given after chemotherapy in 36 patients, and at progression in 18 patients. Study design and inclusion criteria have been published previously (Ferreri *et al*, 2009). The determination of MTX serum levels was performed before and immediately after the end of drug infusion, and repeated every 24 h until the MTX serum concentration fell under the threshold concentration of 0.05 *μ*mol l^−1^. The concentration data of MTX – collected at 0, 24, 48, 72 and 96 h from drug infusion – during the first course of chemotherapy were considered for PK analysis. Leucovorin rescue started 24 h after the start of HD-MTX infusion, administered at a dose of 15 mg m^−2^ intravenous push every 6 h for 12 times or more until MTX serum levels were undetectable. After 48 h from MTX infusion, leucovorin rescue was modified according to MTX serum levels.

### Population PK analysis

Population PK analysis was performed using the nonlinear mixed-effects modelling program (NONMEM) version VI (double precision, level 1.1) (Beal and Sheiner, 1998). First, a basic PK model was developed for MTX concentration–time data. Model selection was based on the minimum value of objective function, the precision of parameter estimates and the fit of the model to the data as approached by graphical plots. Inter-individual variability was estimated using a proportional error model. Second, the following covariates were tested for their relationship with CL_MTX_: patient gender, age, body-surface area (BSA), creatinine clearance (CL_CREA_ according to the Cockroft–Gault formula, assessed before the start of MTX infusion and capped at 140 ml min^−1^), as well as co-medication with anticonvulsant drugs and steroids. Forward selection and backward elimination were used for covariate testing, with a significance level of *P*<0.01.

### Toxicity and response assessment

Adverse events were separately assessed for each chemotherapy course and graded according to the NCI-NCIC CTC version 3.0 (Trotti *et al*, 2003). The worst toxicity per organ and per patient was considered for analysis. Treatment response was assessed on contrast-enhanced brain MRI performed within 7 days before chemotherapy and repeated after the second and fourth treatment cycle and after WBRT. Response definition was based on changes in tumour size of enhanced lesions on T1-weighted MRI, and following the NCI standardised response criteria (Cheson *et al*, 1999). In cases with concomitant positive CSF, cytology examination was performed after the second and fourth treatment cycle and after treatment completion. A reduction of >50% of cell number was considered PR, whereas a lower reduction was considered SD. The maximum response recorded from treatment start was considered for activity analyses. Follow-up examinations were conducted as reported previously (Ferreri *et al*, 2003a).

### Statistical analysis

Individual AUC_HD-MTX_ was compared between treatment groups, patient gender and treatment response using Student's *t*-test. Patients were categorised into those having no response to chemotherapy (SD, PD) and responders (CR or PR). Analysis of variance (ANOVA) was used to compare individual AUC_HD-MTX_ with treatment-associated toxicity. To assess any potential relationship between AUC_HD-MTX_ and clinical outcome, the former was categorised into tertiles, with the higher tertile corresponding to an AUC >980 *μ*mol l^−1^ h and the lower tertile corresponding to an AUC <830 *μ*mol l^−1^ h. Both EFS and overall survival (OAS) were calculated per AUC_HD-MTX_ category using survival analysis and log-rank test, respectively. The following potential predictors for chemotherapy response were studied using multivariate logistic regression analysis: AUC_HD-MTX_ (tertiles), gender, categorical IELSG prognostic score (Ferreri *et al*, 2003b) (0–1, 2–3, 4–5 points) and treatment group. The following potential predictors for clinical outcome (EFS, OAS) were studied using multivariate Cox hazards modelling: gender, IELSG score, treatment group and AUC_HD-MTX_. Both EFS and OAS curves were calculated using the Kaplan–Meier method, and the log-rank test was used to detect potential differences per AUC_HD-MTX_ category. A previously described threshold of AUC_HD-MTX_ ⩾1100 *μ*mol l^−1^ h (Ferreri *et al*, 2004) was additionally analysed on chemotherapy response and clinical outcome. All tests of significance were two-sided; *P*<0.05 was considered significant. All statistical analyses were performed using STATA 10.1 software (STATA Corp, College Station, TX, USA).

## Results

### Patient population and data set

Patient characteristics have been described previously (Ferreri *et al*, 2009). Out of the 79 patients, 55 had available PK data of HD-MTX and were included into this analysis with the following characteristics: median age 56 years, 32 female (58%) and 23 male (42%); 30 patients were randomised to receive HD-MTX and 25 patients to combined HD-MTX/HD-AraC, with a median IELSG-score of 2. Patient characteristics of the PKPD subgroup and of the total population were not significantly different. After chemotherapy, 7 HD-MTX and 18 HD-MTX/HD-AraC patients achieved a CR (18 *vs* 46% *P*=0.006); 9 MTX and 9 MTX/AraC patients achieved a PR, for an ORR of 40 and 69%, respectively (*P*=0.009). At a median follow-up of 30 months, 31 MTX and 23 MTX/AraC patients experienced failure (PD, relapse, death), with a 3-year EFS of 21 and 38%, respectively (*P*=0.01). In all, 12 MTX and 20 MTX/AraC patients are alive, with a 3-year OAS of 32 and 46%, respectively (*P*=0.07).

### Population PK model

**Figure 1:**
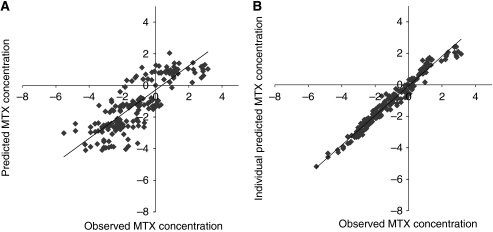
Goodness-of-fit plots of the final population pharmacokinetic model (all data log-transformed, drug concentration as *μ*mol l^−1^). Observed MTX concentrations *vs* model predictions (**A**) and *vs* individual Bayesian predictions (**B**).

The MTX concentration–time data were best described by a linear two-compartment model with first-order elimination from the central compartment. The MTX clearance was 14.9 l h^−1^ (relative s.e. 9.95), with an inter-individual variability of 22.3% and a residual variability of 31.8%. Volume distribution was 71.9 l (±51.5), with an inter-individual variability of 30.1%. Inter-compartmental clearance was 11.2 l h^−1^ (±5.2), with an inter-individual variability of 35%. Two patients had a CL_HD-MTX_ >20 l h^−1^, six patients <7 l h^−1^. Median AUC_HD-MTX_ was 931 *μ*mol^*^l^−1^ h (range 486–1710 *μ*mol^*^l^−1^ h). The AUC_HD-MTX_ was <750 *μ*mol l^−1^ h in 11 out of 55 cases with available PK data (20%), and >1100 *μ*mol l^−1^ h in 12 cases (22%). Creatinine clearance was the only significant covariate on CL_HD-MTX_, resulting in Equation 1 to describe CL_HD-MTX_ as a function of CL_CREA_ (95 ml min^−1^ is the median CL_CREA_ as found in the study group): 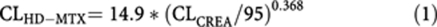


The inclusion of patient age, BSA or concurrent administration of HD-AraC did not improve the model fit. The goodness-of-fit plots supported a good data fit of the final model (Figure 1).

### Statistical analysis

**Figure 2:**
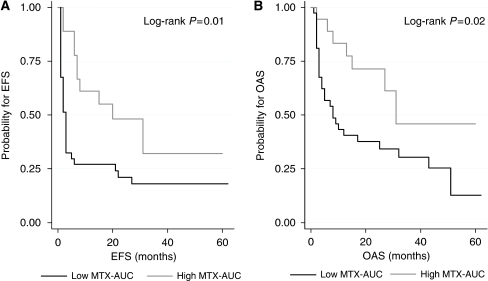
Kaplan–Meier plots for event-free survival (**A**) and overall survival (**B**) grouped according to the highest AUC_HD-MTX_ tertile (>980 *μ*mol^*^l^−1^ h) and the lower two tertiles of AUC_HD-MTX_ (<980 *μ*mol^*^l^−1^ h).

The AUC_HD-MTX_ tertiles are outlined across chemotherapy response and clinical outcome in Table 1. The AUC_HD-MTX_ was not significantly different between treatment groups (902 *μ*mol^*^l^−1^ h in the HD-MTX group, 965 *μ*mol^*^l^−1^ h in the HD-MTX/HD-AraC group, *P*=0.16). The AUC_HD-MTX_ was significantly higher in the 29 responding patients compared with the 26 cases without chemotherapy response (1075 *vs* 867 *μ*mol^*^l^−1^ h, *P*=0.0001). Predictors of a favourable treatment response are outlined in Table 2. Patients with AUC_HD-MTX_ >1100 *μ*mol^*^l^−1^ h had an odds ratio of 3.5 for having a favourable treatment response (*P*=0.03). There was no significant relationship between AUC_HD-MTX_ and toxicity, with the exception of liver dysfunction (AUC_HD-MTX_ 1047 *μ*mol^*^l^−1^ h in patients with any treatment-associated liver dysfunction *vs* 932 *μ*mol^*^l^−1^ h in those without liver dysfunction, *P*=0.02) and neutropaenia (AUC_HD-MTX_ 1036 *μ*mol^*^l^−1^ h in patients with grade 3 or 4 neutropaenia *vs* 914 *μ*mol^*^l^−1^ h in those with no or grade 1 or 2 neutropaenia, *P*=0.007). Patients with the highest AUC_HD-MTX_ exhibited a significantly better EFS and OAS as compared with patients in the lower two tertiles of AUC_HD-MTX_ (Table 2 and Figure 2). The AUC_HD-MTX_ >1100 *μ*mol^*^l^−1^ h was associated with a better EFS and OAS by the log-rank test (*P*=0.023 and *P*=0.056, respectively). Both the AUC_HD-MTX_ and the IELSG score were significant predictors of EFS and OAS using multivariate Cox regression analysis (Table 3). When AUC_HD-MTX_ >1100 *μ*mol^*^l^−1^ h was introduced into Cox regression analysis as a binary covariate, statistical significance was retained (HR=0.51, *P*=0.033 for EFS, HR=0.50, *P*=0.044 for OAS). No association was found between the volume of distribution, inter-compartmental clearance and any of the clinical end points.

## Discussion

This PKPD analysis of HD-MTX in patients from the IELSG no. 20 trial is of special value, as this is the first prospective, randomised trial in PCNSL with completed accrual (Ferreri *et al*, 2003a). This study shows that AUC_HD-MTX_ is the most important and independent predictor of clinical outcome in this group of patients. This is an important issue considering the fact that the HD-MTX/HD-AraC combination is the new standard therapeutic approach to patients with PCNSL (Ferreri *et al*, 2009). Interestingly, this study showed that nearly 75% of patients did not achieve an AUC_HD-MTX_ >1100 *μ*mol^*^l^−1^ h, which has been previously reported as an independent predictor for improved clinical outcome (Ferreri *et al*, 2004). The present results cannot be interpreted as a lack of benefit from combined HD-MTX/HD-AraC treatment, as not all patients had available PK data for HD-MTX, and combined HD-MTX/HD-AraC treatment was still a significant predictor for clinical outcome when AUC_HD-MTX_ was dropped from the Cox model. It still indicates that inter-individual disparities in HD-MTX pharmacology have an important role in clinical outcome, and that optimising individual AUC_HD-MTX_ is an important strategy for improving clinical outcome in PCNSL. Thus, the encouraging results of the IELSG no. 20 trial (Ferreri *et al*, 2003a) might be further improved by individualised MTX administration aimed to achieve a target AUC_HD-MTX_ of 1000 *μ*mol^*^l^−1^ h. This statement is also endorsed by the fact that there was no relevant impact of AUC_HD-MTX_ on drug toxicity. The strengths of this study include a homogeneous patient population, the availability of detailed response, outcome and toxicity data in all patients, first-course PK data of MTX in most patients, as well as population PKPD analysis of HD-MTX time–concentration data. The main limitations of this study are that drug interactions between HD-MTX and HD-AraC could only indirectly be studied because no PK data of HD-AraC were available, and PK data of HD-MTX from later courses were not available. However, the fact that tumour response was usually seen within the first two courses of chemotherapy (Ferreri *et al*, 2009) does suggest a strong correlation between first-course PK data and clinical outcome.

In a retrospective study (Ferreri *et al*, 2004), PCNSL patients treated with MTX-based chemotherapy and an AUC_HD-MTX_ >1100 *μ*mol^*^l^−1^ h showed significantly better response and survival rates. In the IELSG no. 20 trial, only 22% of patients achieved this AUC_HD-MTX_, suggesting room for improving HD-MTX administration. Importantly, a suboptimal AUC_HD-MTX_ was obtained equally in both treatment arms in the IELSG no. 20 trial. Therefore, the introduction of a personalised dose of HD-MTX, according to patient age, gender and CL_CREA_, has the potential to significantly improve treatment activity in these patients, and should be investigated in future trials. According to the present observation, personalisation of the MTX administration schedule should not consider the concomitant use of HD-araC, as the addition of this drug did not change MTX PK. However, this has to be taken with caution, considering the potential for increased toxicity with the combined use of HD-MTX/HD-AraC.

In conclusion, individualised dosing of HD-MTX might have the potential to improve clinical outcome in patients with PCNSL, even when administered concurrently with HD-AraC. In the future, this could be carried out by using first-cycle PK modelling with determination of potential dose adaptations for later cycles using Bayesian analysis.

## Figures and Tables

**Table 1:** Comparison between AUC_HD-MTX_ tertiles, chemotherapy response and clinical outcome

	**AUC_low_ (<830 *μ*mol l^−1^ h)**		**AUC_mid_ (830–980 *μ*mol l^−1^ h)**		**AUC_high_ (>980 *μ*mol l^−1^ h)**		
**Covariate**	**Pts (%)**	**OR (95% CI)**	**Pts (%)**	**OR (95% CI)**	**Pts (%)**	**OR (95% CI)**	***P*-value**
*ORR*							
SD/PD	14 (54)	Ref	10 (39)	Ref	2 (7)	Ref	
CR/PR	5 (17)	0.18 (0.05–0.61)	8 (28)	0.61 (0.19–1.89)	16 (55)	14.7 (2.93–74.4)	<0.001^a^
							
	**Pts (%)**	**%**	**Pts (%)**	**%**	**Pts (%)**	**%**	
*Outcome*							
3-Year EFS	19 (34)	21.1	18 (33)	20.8	18 (33)	32.1	0.04^b^
3-Year OAS	19 (34)	30.7	18 (33)	30.5	18 (33)	46.0	0.06^b^

Abbreviations: AUC_HD-MTX_=area under the curve of high-dose methotrexate; Pt=patient; OR=odds ratio; CI=confidence interval; ORR=objective response rate; SD=stable disease; PD=progressive disease; CR=complete response; PR=partial response; Ref=reference; EFS=event-free survival; OAS=overall survival.

a Analysis of variance.

b log-rank test.

**Table 2:** Predictors for chemotherapy response (complete and partial remission) using multivariate regression modeling

**Covariate**	**OR**	**95% CI**	***P*-value**
*Patient gender*			
Male	Ref		
Female	0.48	0.08–2.92	0.42
			
*IELSG risk score*			
0–1	Ref		
2–3	0.05	0.005–0.44	0.007
4–5	0.03	0.002–0.48	0.01
			
*Treatment group*			
HD-MTX	Ref		
HD-MTX/HD-AraC	9.33	1.33–65.53	0.02
			
*AUC*_*HD-MTX*_ *(tertiles, μmol l*^*−1*^* h*^*−1*^*)*			
<830	Ref		
830–980	5.21	0.73–37.3	0.10
>980	121.9	7.80–190.1	0.001

Abbreviations: OR=odds ratio; CI=confidence interval; IELSG=International Extranodal Lymphoma Study Group; HD-MTX=high-dose methotrexate; HD-AraC=high-dose cytarabine; AUC=area under the curve; Ref=reference.

**Table 3:** Predictors for event-free and overall survival using multivariate Cox regression analysis

**Covariate**	**HR**	**95% CI**	***P*-value**
*Event-free survival*			
Patient gender			
Female	Ref		
Male	1.12	0.52–2.40	0.77
			
IELSG score			
0–1 *vs* 2–3 *vs* 4–5 points	1.71	1.04–2.81	0.03
			
Treatment group			
HD-MTX	Ref		
HD-MTX/AraC	0.65	0.34–1.25	0.19
			
AUC_HD-MTX_			
Per 100 *μ*mol l^−1^ h increase	0.82	0.69–0.98	0.03
			
*Overall survival*			
Patient gender			
Female	Ref		
Male	1.77	0.76–4.10	0.19
			
IELSG score			
0–1 *vs* 2–3 *vs* 4–5 points	1.82	1.00–3.31	0.05
			
Treatment group			
HD-MTX	Ref		
HD-MTX/AraC	0.80	0.39–1.65	0.54
			
AUC_HD-MTX_			
Per 100 *μ*mol l^−1^ h increase	0.73	0.59–0.89	0.002

Abbreviations: HR=hazard ratio; CI=confidence interval; IELSG=International Extranodal Lymphoma Study Group; HD-MTX=high-dose methotrexate; HD-AraC=high-dose cytarabine; AUC=area under the curve; Ref=reference.
